# Integrating Face Washing into a School-Based, Handwashing Behavior Change Program to Prevent Trachoma in Turkana, Kenya

**DOI:** 10.4269/ajtmh.19-0205

**Published:** 2019-08-05

**Authors:** James B. Tidwell, Cristin Fergus, Anila Gopalakrishnan, Esha Sheth, Myriam Sidibe, Leah Wohlgemuth, Avinish Jain, Geordie Woods

**Affiliations:** 1 Harvard Kennedy School of Government, Cambridge, Massachusetts;; 2 London School of Economics and Political Science, London, United Kingdom;; 3 Unilever PLC, London, United Kingdom;; 4 Sightsavers, London, United Kingdom

## Abstract

Trachoma is the leading infectious cause of blindness, and facial cleanliness is associated with reduced odds of trachomatous inflammation and *Chlamydia trachomatis* infection, but there is little evidence of how to drive this behavior change at scale. We report the results of a program integrating face washing into a school-based handwashing promotion program in Turkana County, Kenya. Children aged 5–15 years participated in an intervention delivered to schools in two phases, along with a third phase receiving the intervention after the evaluation, which served as a control. The primary outcome was the number of face washing events that took place when handwashing occurred, which was measured by a 3-hour structured observation at all 67 schools, and a total of 3,871 handwashing events were observed. Differences in observed in face washing behavior between each phase and the control schools were calculated using log-binomial regression with clustering at the school level, whereas survey responses on knowledge of trachoma transmission and prevention were compared using χ^2^ tests adjusted for clustering at the school level. Face washing during handwashing events was higher in schools after 12 months (59.3%) and 20 months (44.2%) than in control schools (18.7%, *P* < 0.001). Trachoma knowledge was higher in schools evaluated after 12 months (80%) and 20 months (70%) than in control schools (42%, *P* < 0.001), and knowledge of some of key preventive behaviors was higher in intervention schools. Integrating face washing messages into school-based handwashing promotion programs increased face washing, which may help to prevent trachoma when combined with other interventions.

## INTRODUCTION

Blinding trachoma is a condition of the eye caused by repeated *Chlamydia trachomatis* infections and is the leading infectious cause of blindness globally.^1^ About 158 million people live in areas with endemic trachoma, primarily in sub-Saharan Africa, the Middle East, and Asia.^2^ About 2.2 million people are visually impaired to some degree because of trachoma, and 1.2 million are permanently blind.^3^

The WHO called for the elimination of Trachoma as a public health problem through the founding of the Global Alliance for the Elimination of Blinding Trachoma by 2020 (GET2020) strategy in 1993.^4^ The WHO recommends the “SAFE” strategy for elimination, consisting of surgery, antibiotic treatment, facial cleanliness, and environmental sanitation. Significant progress has been made in reducing the effects of trachoma globally, with the number at risk dropping between 2007 and 2018 from 1.2 billion^5^ to 158 million.^6^ Although considerable evidence exists to support the effectiveness of surgery to reverse turned-in eyelashes^7^ and mass annual antibiotic treatment,^8^ there is less certainty on the impact of environmental sanitation^9^ on the proper definition, measurement, or impact of facial cleanliness.^10^

Face washing is an integral component of the SAFE strategy for reducing the amount of ocular and nasal discharge present on the face.^11–13^ However, assuming that face washing may significantly reduce the transmission of trachoma, there is a need for more evidence-based interventions that effectively and sustainably promote face washing.^14,15^ The recommended behavior is commonly to wash the face with soap and water when dirty and let the face dry, but increasing the performance of preventive health behaviors in general is hard, especially when perceived risks of failing to perform the behavior are low.^16^

Trachoma is one of a group of diseases known as neglected tropical diseases (NTDs), which have historically received less attention in health promotion efforts. However, because of the many synergies between preventive behaviors for diseases related to poor water, sanitation, and hygiene (WASH), and NTDs, increasing efforts are being made to advocate for integrating preventive NTD messages into WASH programs.^17,18^ The WHO recently called for addressing the determinants of NTDs through WASH programming and joint policy frameworks.^19,20^ Some diseases, such as soil-transmitted helminthiasis, are prevented by performing exactly the same behaviors (in this case, reducing open defecation, maintaining sanitation facilities, water treatment, handwashing with soap, and food hygiene) as are typically recommended by WASH programs.^17^ Others require different but related behaviors, such as washing the body to reduce acute attacks of lymphatic filariasis, which requires sufficient quantities of water be present to perform the washing.

Recently, there have been calls to think through face washing programs for the elimination of trachoma using available behavior change theories, with a particular focus on the sustainability of behavior change approaches.^14,15^ One promising possibility is integrating face washing into handwashing with soap. Handwashing with soap is one of the most important, cost-effective health behaviors to promote^21^ as it significantly reduces rates of diarrheal and respiratory disease and has many other benefits including increased rates of school attendance^22–24^ and has also been linked with reducing the prevalence of trachoma.^25^ It is frequently recommended that handwashing with soap be performed at five key public health occasions: after defecation or cleaning a child who has defecated, before cooking meals, before feeding a child, and before eating.^22–24^

Adding face washing to this routine is promising for several reasons. First, the behaviors require the same materials (soap and water) and so can occur in the same setting. Settings drive many behaviors and have been found key to driving handwashing behavior. Second, when lathering up one’s hands with soap or washing it off with water, one can easily also perform the analogous behaviors for washing the face. Integrating similar behaviors into unified routines is likely to result in increased rates of uptake of the desired behavior.^26^ Third, handwashing with soap is a widely promoted practice, and so has the potential to reach a large audience with face washing messages.

Behavior change programs integrated into schools have great potential to drive sustainable behavior change in children if they can be delivered by teachers or other built-in mechanisms as they reach children in a setting where there is less supervision (compared with, say, parental supervision before meals in the home), they can leverage positive peer pressure, and they allow all children in schools the opportunity to practice healthy behaviors regardless of home environment. We report here the results of integrating a face washing promotion program^27^ into a widely conducted, school-based handwashing promotion program (the School of Five^28^). The main objective of the study was to provide evidence for a program to promote face washing in a cost-effective manner at scale in endemic areas for trachoma. The key research question was whether face washing could be integrated into an existing handwashing promotion program in a way that led to sustained increases in face washing.

## METHODS

### Setting.

This study took place in Turkana County in northern Kenya, which has the highest burden of active trachoma in the country (42.3% of the population^29^). Turkana County has the highest percentage of the population who live below the poverty line (88%) and have no education (82%), and the second highest rate of unimproved sanitation (92%). Its access to improved water sources (39%) is in the bottom third of Kenyan counties, as the nearby Lake Turkana provides more water than some interior counties can access.^30^

### Study Design.

**Figure 1.**
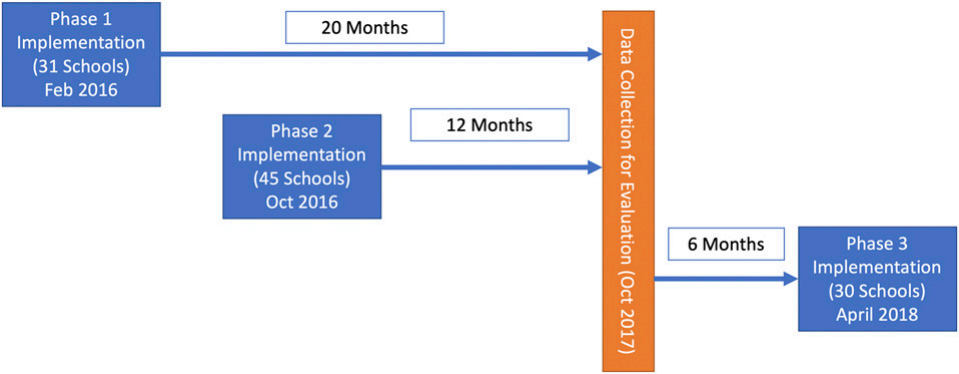
Study design. This figure appears in color at www.ajtmh.org.

The intervention was delivered in 116 government primary schools across four of the seven subcounties of Turkana County from February 2016 to October 2017. The intervention was piloted in 10 schools (results not reported in this study) and then delivered in three phases. Phase 1 was delivered in February 2016 to 31 schools; phase 2, with only small modifications, the intervention (described in the following text) was delivered in October 2016 to 45 schools; and phase 3 was delivered in April 2018 to 30 schools. The fieldwork for this evaluation took place between September 18 and October 6, 2017, so that data from phase 1 (20 months prior) and phase 2 (12 months prior) were compared with schools selected for phase 3, which served as control schools, because they were surveyed before they received the intervention and had experienced no program activities (Figure 1). Intervention schools were randomly selected from phases 1 (17 schools) and 2 (20 schools), and only schools with basic water supplies where catchment areas of the schools did not overlap were included in the sampling frame. Selection of control schools excluded any schools in phase 3 areas that had no toilets, stored water, or handwashing stations. These were deemed the most important factors on which to match, as the implementation area was relatively homogeneous in terms of rural setting, ethnic makeup, and socioeconomic status.

### Sample selection and data collection.

For each selected school, data were collected from four different sources. First, one class from levels 1–3 and one class from levels 4–8 were randomly selected from each school and 10 children per class were randomly selected for a school-aged child questionnaire to assess knowledge of trachoma and other information communicated by the program and collect demographic information. Second, each school was assessed using a school audit tool to collect information about the WASH infrastructure at the school and the presence of program materials (such as posters) to evaluate the delivery and sustainability of the intervention. Third, a structured observation tool was used to record directly observed handwashing and face washing behavior using school WASH facilities for one 3-hour observation per school. One staff member performed these observations discretely while other staff members were performing the school audit and administering school-aged child questionnaires. Observers noted the type of washing event, the occasion (e.g., before eating food and after using the latrine), if and how hands and face were washed, and whether anyone assisted the child in washing. Finally, a household questionnaire was given to eight randomly selected caretakers from households with a school-aged child in two randomly selected villages in the catchment area of each school. The household questionnaire captured household demographics, assets, and knowledge, beliefs, attitudes, and self-reported behavior for handwashing and face washing.

Data collection tools were developed by Sightsavers staff members with technical support from Lifebuoy based on tools used in prior evaluations. A team consisting of an evaluation coordinator, three supervisors, and 20 surveyors were given a 3-day training to understand the objectives of the evaluation and the tools to be used. Teams pretested school- and household-based questionnaires and met in a joint feedback session to revise the tools where any clarifications to language or procedures were needed. Role playing and field exercises were performed in two schools and villages not selected for the survey, and review from a Sightsavers technical advisor found a 96% concordance between survey items. Data collection was performed with paper forms, which were double entered into a Microsoft Access database by Sightsavers staff in Nairobi, Kenya. Electronic data were stored on password-protected computers, and paper forms were stored in locked cabinets.

### Intervention content.

The Super School of Five program was based on Unilever-Lifebuoy’s School of Five program, which trains teachers to deliver hygiene promotion messages based on the key principles of awareness, commitment, reinforcement, and reward to promote handwashing with soap before breakfast, lunch, and dinner; after defecation; and during bathing.^22^ The program was designed and delivered by Lifebuoy, a soap brand of Unilever whose social mission aims to increase handwashing with soap behaviors among schoolchildren and mothers, in partnership with Sightsavers, an organization that has been working on NTDs such as trachoma since 1950. This program targeted children aged 5–15 years in schools because young children are most at-risk for active trachoma, and caretakers, such as older siblings, are an important route of transmission because of close contact with young children.^31^ Schools appointed two “champion teachers” to oversee implementation and select and train students, called “little ministers,” to ensure that water and soap were available and that their classmates used the written diaries. Champion teachers were trained by Sightsavers and Unilever Lifebuoy staff, and in turn trained other teachers in their schools and worked with the school administration to develop sustainability plans to ensure soap and water were always available and that the behavior change was sustained in the long term. The “champion teachers” trained other teachers to deliver a 21-day campaign that raised awareness through interactive stories on a flipchart, games, songs, and graphic novels. Students made a public commitment to wash their hands during an assembly or in classrooms at the beginning of the campaign, received reinforcement by recording behavior in a written diary (used as a tool to encourage habit formation, not a source of data for the evaluation), and enrolled other members of the community to pass on critical messages. Soap was provided to the schools during this 21-day campaign only, with a committee in each school tasked with ensuring future provision. Teachers and students receive a certificate of completion as a reward at the end of the program, and well-performing schools were rewarded with a water storage tank.

Trachoma messages were integrated into the curriculum by encouraging face washing at each handwashing occasion. One of the superheroes in the graphic novel who promoted bodily cleanliness (soap use while bathing) was also altered to include face washing as a way to be clean and socially presentable. An additional lesson was also added to explain trachoma and the importance of face washing. Messages and materials were developed by Sightsavers and Lifebuoy in consultation with international experts in hygiene and trachoma behavior change and tested in the 10-school pilot in 2014, with separate focus group discussions with primary school students, teachers, and caregivers to understand clarity and acceptability of the intervention. Specific messages on motivating behavior change targeted disgust as a key driver, which has been shown to be effective for handwashing.^32,33^

After phase 1, several implementation challenges were noted, with small tweaks introduced in phase 2. First, there was low turnout of parents at community-level meetings, and so small soap samples were given at these meetings as an incentive to attend. Second, a higher-than-anticipated number of champion teachers and little ministers missed initial trainings, but this was largely because of an ongoing drought and teacher strikes, which were no longer a problem in phase 2. Third, graphic novels with superhero stories used to convey trachoma messages in phase 1 were too challenging for younger children to read, and so for phase 2, these were adapted into larger format posters placed on each classroom wall so that teachers could also use them as a group-learning tool with students.

### Study outcomes.

As the primary research question was whether a face washing behavior could be added to a handwashing routine, the primary outcome of the study was the percent of times that face washing occurred during handwashing events. Additional outcomes of interest included the percentage of handwashing occasions where proper handwashing behavior (using soap) and/or face washing behavior (using soap: drying with a clean towel or allowing to air-dry) occurred, changes in knowledge related to trachoma transmission and preventive behaviors, and measures of the fidelity of program delivery.

### Data analysis.

For the primary outcome of proportion of handwashing events where face washing occurred, log-binomial regression was used to estimate risk ratios associated with exposure to the program with errors clustered at the school level. Proportions of handwashing and face washing events where behaviors were properly practiced were both analyzed similarly. Levels of knowledge achieved by the educational intervention were compared using χ^2^ tests adjusted for clustering at the school level using Donner’s method.^34^ Process indicators were also analyzed separately using simple descriptive statistics to understand how well the program was delivered and the levels of sustainability achieved. All data analyses were carried out with R version 3.5.0 (R Core Team, Vienna, Austria).

### Ethical procedures.

Key stakeholder workshops were conducted with national leaders, country health officials, and community leaders with approvals sought formally and informally. Consent was initially sought from head teachers at each school. They subsequently informed parents, the school committee members, and students about the study, and the parents or guardians of each child were free to refuse for their child to participate in the study. On the day of the school-aged child survey, children being surveyed were informed about the survey procedures and told that their participation was voluntary and that they could opt out at any time. Individual written consent was obtained from each child, and children who could not write indicated consent with a thumb print. Names of participants or other personally identifying information were not recorded. Household surveys addressed to caretakers of school-aged children followed similar procedures. Ethical approval was obtained from the Amref Ethics and Scientific Review Committee (Ref: AMREF – ESRC P367/2017) and the Turkana County Director of Education (Ref: TUR/CDE/CONF/1/VOL.1/17).

## RESULTS

School-aged child surveys, school audits, and behavioral observations took place at 67 schools, with 17 from phase 1, 20 from phase 2, and 30 control schools (Table 1), and 1,340 household surveys were conducted (Table 2). Results are displayed in each table for control (which had received no intervention at the time of data collection), phase 1 (evaluated 20 months after delivery), and phase 2 (evaluated 12 months after delivery) groups, respectively, based on the assumption that the effects of the intervention decayed over time. Although almost all audited schools had water present (35 in the intervention and 26 in the control), only six schools from the intervention and one from the control had soap present at the time of the audit. We therefore did not analyze the prevalence of properly performed hand or face washing behavior with both soap and water because of the small sample size and possibility of bias introduced by a small number of schools with soap present. Whereas most intervention schools had designated handwashing facilities present, only half of control schools did so, meaning that overall rates of handwashing were likely affected by infrastructure present at each school.

**Table 1:** School audit results from end line evaluation

Variable	Control	Phase 2 (12 months after delivery)	Phase 1 (20 months after delivery)
*n*	30	20	17
Water connection present (*n* [%])	26 (87%)	18 (90%)	17 (100%)
Designated handwashing facilities present (*n* [%])	15 (50%)	20 (100%)	13 (76%)
Soap present at handwashing facility (*n* [%])	1 (3%)	5 (25%)	1 (6%)
Latrine present (*n* [%])	28 (93%)	20 (100%)	17 (100%)

**Table 2:** School-aged child survey results from end line evaluation

Variable	Control proportion (95% CI)	Phase 2 (12 months after delivery) proportion (95% CI)	Phase 1 (20 months after delivery) proportion (95% CI)
*n*	600	400	340
Age (mean)	7.8	7.7	8.2
Has heard of trachoma	41.5% (39.6%, 43.4%)	80.0% (78.4%, 81.6%)*	70.1% (67.9%, 72.3%)*
Trachoma is transmitted by			
Flies	41.4% (39.5%, 43.3%)	46.8% (44.4%, 49.2%)	42.6% (40.0%, 45.2%)
Contact with infected person	3.3% (3.0%, 3.6%)	13.0% (11.9%, 14.1%)	10.3% (9.3%, 11.3%)
Using dirty cloth of infected person	8.9% (8.3%, 9.5%)	18.8% (17.3%, 20.3%)	14.9% (13.6%, 16.2%)
Evil spirits	2.8% (2.6%, 3.0%)	1.3% (1.2%, 1.4%)	0.4% (0.4%, 0.4%)
Knows that trachoma may be prevented with			
Handwashing	37.7% (35.8%, 39.6%)	71.1% (69.1%, 73.1%)*	55.1% (52.5%, 57.7%)*
Face washing	57.5% (55.5%, 59.5%)	73.5% (71.6%, 75.4%)*	61.6% (59.1%, 64.1%)
Latrine use	5.4% (5.0%, 5.8%)	19.9% (18.3%, 21.5%)*	13.3% (12.1%, 14.5%)*
Using clean towels	19.1% (17.9%, 20.3%)	7.8% (7.1%, 8.5%)	10.9% (9.9%, 11.9%)
Taking medicine	3.3% (3.0%, 3.6%)	1.5% (1.4%, 1.6%)	3.8% (3.4%, 4.2%)

* *P* < 0.001 compared with control.

The intervention was associated with more respondents having heard of trachoma (80% phase 2, 70% phase 1 versus 42% control), but no differences in knowledge of how trachoma is transmitted were found (Table 2). However, knowledge of preventive behaviors was higher, with phase 2 respondents reporting higher knowledge of handwashing, face washing, and latrine use to prevent trachoma and phase 1 respondents still reporting higher knowledge of handwashing for prevention.

The intervention was associated with a large increase in face washing during handwashing events (Table 3). Face washing rates per handwashing event were higher 12 months after intervention (59%) than after 20 months (44%), but both groups had higher rates of performing both behaviors together, with risk ratios of 3.17 and 2.36, respectively. These handwashing events were often occurring at times of public health significance (between 67% and 87% across all phases occurred before eating or after using the toilet), although no denominator capturing the number of key public health occasions was recorded. Although the use of soap was not analyzed, the second aspect of proper face washing behavior to prevent trachoma (allowing to dry off using nothing or a clean cloth) was performed at high rates across both phases and the control (97.9% in the control, 96.2% in phase 1, and 96.3% in phase 2).

**Table 3:** Intervention impact on primary outcome of face washing at handwashing occasions

Phase	Face washing/total observations	Proportion of hand washes with face washing (95% CI)	Risk difference (95% CI)	Risk ratios (95% CI)
Control	61/326	18.7% (14.5, 22.9)	ref	ref
Phase 2	1,469/2,476	59.3% (57.4, 61.3)*	40.6% (36.0, 45.3)*	3.17 (2.45, 3.90)*
Phase 1	472/1,069	44.2% (41.2, 47.1)*	25.4% (20.3, 30.6)*	2.36 (1.80, 2.92)*

* *P* < 0.001.

**Figure 2.**
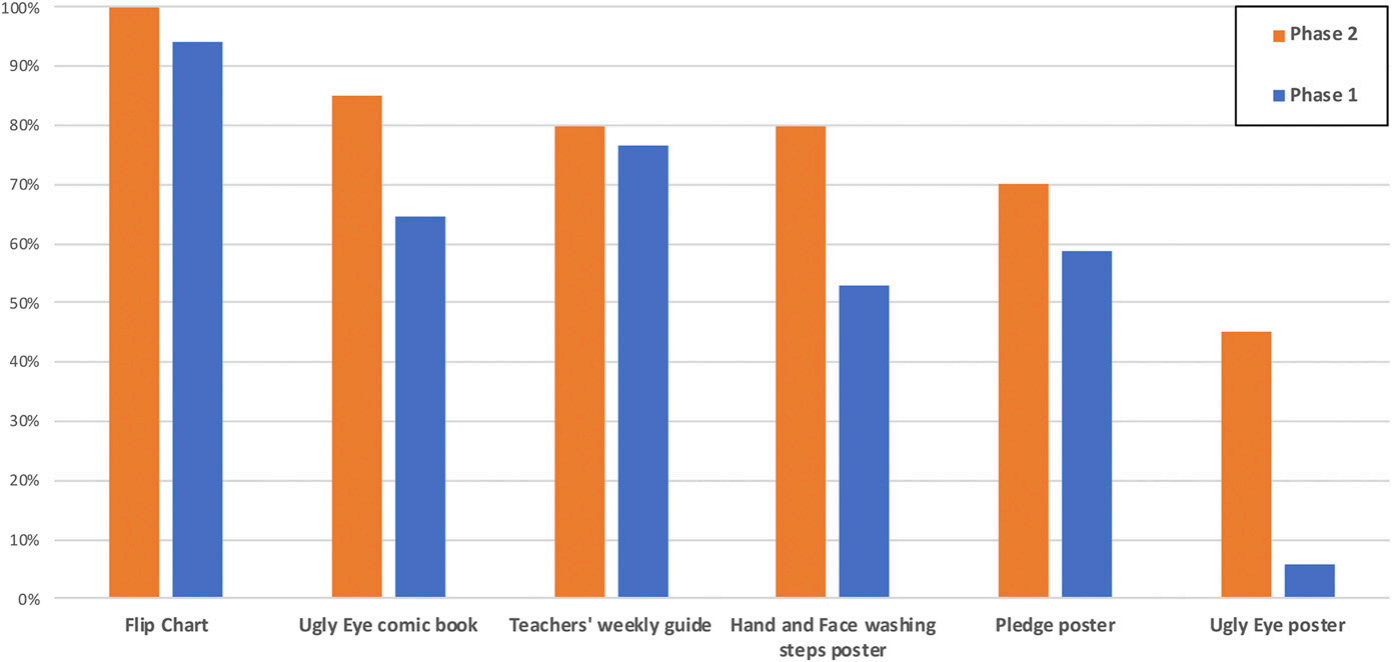
Presence of intervention materials in schools. This figure appears in color at www.ajtmh.org.

The effectiveness of the intervention delivery and the sustainability of results were assessed in several ways (Figure 2). First, the school-aged child questionnaire found that 86% of children from phase 2 and 79% from phase 1 recalled being in a hygiene and trachoma program. About half remembered using the diary to record handwashing and face washing behavior (55% from phase 2 and 53% from phase 1). About a third of students recalled being taught about face washing, either remembering that it should be performed with soap and water (20.3% phase 2 and 19.1% phase 1) or only with water (12.0% phase 2 and 11.5% phase 1). Program materials used for continued instruction were present in most schools, with flip charts (100% phase 2 and 94% phase 1) being the most common. Posters were also still present in many schools, with handwashing and face washing steps (80% phase 2 and 53% phase 1), and pledge posters (70% Phase 2, 59% Phase 1) more common than ugly eye posters (45% phase 2 and 6% phase 1), which were used to instruct younger children, but that also took up considerably more space.

## DISCUSSION

The rates of face washing when performing handwashing were significantly higher in schools that received the Super School of Five intervention than similar schools that did not receive the intervention, suggesting that integrating face washing behavior into a handwashing routine may be an effective way to increase rates of face washing among children. The intervention was associated with higher reported knowledge of trachoma and of steps taken to prevent it, although knowledge of the causes of transmission were not different between intervention and control arms. It is unclear whether strengthening the intervention to increase retention of this knowledge would result in a larger effect, but such knowledge is clearly not necessary to see significant changes in behavior. These increases in face washing during handwashing events were sustained even over a 20-month time frame. The intervention sought to institutionalize sustainability through training teachers, providing teaching and promotional materials, and encouraging the development of a sustainability plan, and this approach seems to have been effective.

These results suggest that achieving the “F” (face washing) of the WHO’s SAFE strategy for trachoma prevention may be possible in-part through school-based programs, reducing the prevalence of the leading cause of blindness globally.^1^ Rates of face washing behavior increased significantly, and drying by using a clean cloth or allowing to air dry was performed in almost all cases. However, to see sustainable and widespread public health gains, significant consideration should be given to how to ensure the availability of soap for handwashing and face washing in schools, which is a very different kind of behavior change challenge. Another consideration suggested by the experience of delivering this program is that although seasonality may be an important consideration for WASH program evaluation, delivery of school-based programs as stepped-wedge-style interventions^35^ may help to capture the variability in program effects seen when scaling the program or clarify the ideal time frame for intervention delivery. In this intervention, unexpected teacher strikes and droughts around the time of delivering phase 1 disrupted the program roll out, although the actual impact on results of this challenge is impossible to distinguish from the effects of a longer time between program delivery and evaluation.

A lack of standardized, validated measures of face washing behavior also presents a challenge for interpreting the study results. Handwashing and face washing behaviors were observed for a 3-hour window at schools. Structured observations for short time frames may underestimate rates of face washing behavior and display considerable bias considering face washing plausibly takes place during bathing as well as more general concerns about reactivity to observation.^36^ As soap was not present at many of the schools during the evaluation, it may be that children developed routines of washing their faces with soap at home before or after school. Observing a limited number of hours in the day presents a biased measure when the denominator is “whole days.” Consensus must be reached on valid measures of face washing, similar to work that has been performed for handwashing, with the validity of direct or indirect self-reported measures assessed.^37,38^ This is the subject of several ongoing trials, and failure to find such a measure could represent one of the most significant challenges for integrating WASH and NTD programs.^17^

This study suggests both benefits and challenges to integrating WASH and NTD programs. Although high rates of handwashing with soap can be difficult to attain, incorporating face washing with even low rates of such behavior, regardless of whether it is performed at key public health occasions, may be sufficient to achieve adequate facial cleanliness. However, such integration may not be possible with all kinds of handwashing promotion programs, such as infrastructure-altering interventions^39^; in contexts where knowledge of trachoma and preventive behaviors is low, some education may still be necessary.

There are several limitations of this study that should be noted. First, although there may be selection bias resulting from nonrandom allocation of schools to intervention arms, the differences observed between study arms are sizable enough that it is unlikely that this fully explains the observed effects. As structured observations could not be connected to particular students and few differences in the limited number of assessed school-level confounders were found, an analysis adjusting for observed covariates was not possible. Differences in school handwashing and face washing infrastructure at the time of evaluation may represent a secondary effect of the intervention or preexisting differences between schools. However, although rates of handwashing at key public health occasions may be affected by this discrepancy, we think that rates of face washing during handwashing events are less likely to be affected.

Second, the number of key public health occasions for handwashing (particularly relevant for this study would be after using the toilet and before eating) was not recorded; there was no “denominator” by which to assess the rates of handwashing. Therefore, rates of handwashing and handwashing with soap, soapy water, or ash are only noted in the results and not considered a study outcome. There was also no attendance data collected on the day of the observation, and so no “denominator” for face washing. The recommended behavior of washing the face with soap and water and then drying with a clean cloth or allowing to air dry at least once daily cannot be observed during a limited window during the day without significant potential for bias. This limited the scope of evaluation to assessing how consistently face washing behavior was added to the handwashing routine and properly performed.

Finally, the direct public health effects of the intervention are unclear because of the limited windows of behavioral observation and other measurement challenges along with little clarity on the exact impact of proper face washing behavior. However, the relative ease with which such behaviors may be integrated into existing WASH programs in schools and the plausibility of such an impact implies that it may be advisable to incorporate face washing promotion until the evidence dictates otherwise. In particular, any differences in the rates of handwashing with soap caused by such integration should be evaluated, as this is the most likely source of any reduced effectiveness due to integration.

## CONCLUSION

Integrating face washing into the School of Five program was associated with higher rates of face washing during handwashing events. Despite the need to establish standardized measurement methods for face washing, the effect size was large enough to suggest that the intervention may have a substantial impact on rates of face washing behavior. The integration of trachoma prevention messages into on-going, widespread hygiene promotion programs in schools may result in long-term practice of face washing, which could be effective when used at scale to contribute to the elimination of trachoma as a public health problem.
